# The Use of Online Training Tools in Competition Cyclists During COVID-19 Confinement in Spain

**DOI:** 10.3389/fpsyg.2021.622905

**Published:** 2021-03-18

**Authors:** Antonio Moreno-Tenas, Eva León-Zarceño, Miguel Angel Serrano-Rosa

**Affiliations:** ^1^Department of Behavioral Sciences and Health, Miguel Hernández University, Elche, Spain; ^2^Department of Psychobiology, Faculty of Psychology, University of Valencia, Valencia, Spain

**Keywords:** COVID-19, online training tools, confinement, emotions, cycling psychology

## Abstract

COVID-19 confinement has supposed a challenge to the whole wide world, especially in athletes that have frustrated their expectations about training programs and competitions. Specifically, competition cyclists during confinement had lot of difficulties to train due to the need to train outside their homes. However, the increase of online training sessions, or virtual training tools could help to overcome training difficulties due to confinement although there are not studies that analyse the effects of using these types of tools in cyclists. This study aims to test how the use of online training tools in competition cyclists during confinement is related to training frequency and duration, and emotions. 329 cyclists filled an online questionnaire about feelings during confinement and the use of online training tools, before and during confinement. Results showed that the use of online training tools was associated with higher frequency and duration of trainings. Moreover, those who used these tools felt more positively about their future and preparation to compete, feeling more energy than those who did not used online tools. In addition, cyclists that started using these online tools (including virtual roller training) during confinement increased the frequency of training. In conclusion the use of online training tools for cycling could be appropriate to maintain training levels and energy and positive feelings about their future.

## Introduction

The confinement suffered because of the COVID-19 has had a strong impact on people’s lives as countless studies show reporting high levels of anxiety, depression, distress, or insomnia (Ammar et al., 2020a; Clemente-Suárez et al., 2020; Fu et al., 2020; Maugeri et al., 2020; Rossi et al., 2020; Trabelsi et al., 2021). In the case of athletes, this impact may have been greater as different expectations of competition and training have been frustrated. This has been even more important for athletes who need to train outdoor in which the greatest mental impairment has been observed (Beas Jiménez et al., 2018; Fu et al., 2020).

Cycling is an endurance sport that needs to train outdoors (mainly on the road). It is estimated that world tour professional male cyclists ride, between training and competitions, within 25,000 and 35,000 km per year, while world tour female cyclists ride between 13,000 and 18,000 km per year (Sanders et al., 2019). For this reason, cyclists could have been affected highly negatively by the confinement. However, despite the need to train outdoors, as far as we know there are no studies that have tested the effect of confinement on professional cyclists. Thus, from a practical point of view, cyclists, physical trainers, and teams have had to develop useful alternatives to maintain the level of fitness and motivation.

Along with the increase in psychosocial and emotional disorders during confinement, there has also been an increase in the use of technology for training (Ammar et al., 2020b, 2021a). Thus, thanks to the accessibility of training technology, countless mobile and online applications have emerged to allow training at home. Online apps can guide physical exercise and offer reinforcements and contingencies that improve physical fitness (although, these type of publications are scarce and there is excessive fragmentation of them, according to a recent bibliometric study) (Liu and Avello, 2020). In addition, several studies have suggested the efficacy of exergaming applications to increase adherence to exercise and reduce stress, anxiety and mood disorders during confinement (Bentlage et al., 2020; Chtourou et al., 2020). Thus, the use of digital technology has been recommended to improve adherence to exercise due to its social relevance and impact on health in other types of populations, such as the elderly (Ammar et al., 2021b). Apart from applications for training there are numerous applications in the field of psychological or motivational training (Boudreaux et al., 2018; Oh et al., 2020), sleep monitoring for the recovery and improvement of the athlete’s performance (Gershkovich et al., 2020), reducing the risk of injuries (Halson, 2019) or even for the control of intake in obesity (Keogh et al., 2016). There are some studies that show that the use of these apps can be promising to promote physical activity, quality of life, even self-efficacy in sedentary individuals (Gür et al., 2020; Price et al., 2020) although in a recent systematic review (Truelove et al., 2020) it has been indicated that there are some of those studies with methodological deficiencies that compromise the conclusions, suggesting the need for more rigorous and systematic research.

To our knowledge there is little information about the use of these applications in the case of professional or competition athletes despite the large offer and benefits. In the case of cycling, we have not found any studies investigating whether these apps are used, although a study with people who regularly use the bicycle shows that the use of GPS smartphone apps is associated with a higher frequency of cycling and a greater perception of strength (Angosto et al., 2020). Additionally, in recent years the use by competition cyclists of virtual training simulators that improve performance of conventional rollers has increased. These interactive training tools allow cyclists to virtually compete against each other, monitor their workouts, and even schedule team workouts. They also have functionalities that allow quantifying training loads and physiological adaptations, relevant parameters for any training method, incorporating meters and bluetooth or ANT+ connections for different sensors that allow instant information and data on the main physiological parameters (Mujika, 2017). In this way, cyclists training and performance could be monitored and optimized during indoor training in a similar way to training on the road or in competition (Passfield et al., 2017), with greater efficiency than other instruments such as fitness monitors activity, much cheaper but also much less reliable (Boudreaux et al., 2018). These tools could serve for monitoring the detraining period and quantifying the rate of loss of physical fitness caused by confinement, and planning the lap to normal activity, as it is done in other sports (Eirale et al., 2020). All this could provide an important advantage over non-virtual training (with roller and without interaction), considering the value of social interaction and motivation that they can provide.

In brief, confinement has forced competition cyclists to train indoors and numerous testimonies indicate that many of them have had to start (or use more frequently) to use online training tools (or apps) to maintain training level. However, there is no objective data showing whether this use is effective in improving training frequency or whether it has any effect on the way confinement is experienced. Therefore, the objective of this work is to analyse the use of online training tools and virtual rollers by competition cyclists to confirm the impact of these tools on their perception of confinement, training routines, and mood. This aim has been subdivided into three specific objectives.

First (objective 1) the association between the use of training online tools and the frequency and duration of training during confinement, as well as with mood and to follow training routines will be studied. We expect that the more use of online training tools the higher frequency and duration of training workouts. Moreover, cyclist that employ these online training tools will have better mood and less difficulties following training routines during confinement.

Second (objective 2), those participants who did not use online tools before the confinement will be compared, in the above-mentioned variables, with those who employed them. Complementarily, considering that the use of online training applications may have changed due to confinement, it will be analyzed whether the increase in the use of online training tools during confinement is associated with an increase in frequency and duration of training workouts, mood, and difficulties in following daily routines. In both cases, it is expected that the use of such tools will be associated with the increase the frequency and duration of training, as well with positive mood and less difficulties following routines.

Finally (3rd objective), as virtual rollers suppose greater involvement in training, the use of virtual roller will be studied expecting that cyclists using virtual rollers would train more frequently (and longer training workouts) and will have more positive mood and better competitive perspective during confinement in comparison to those participants not using virtual rollers.

## Materials and Methods

### Participants and Procedure

329 competition cyclists (65 women) with a mean age 32.21 (SD = 11.75) participated in this study. Data collection occurred during the complete confinement (between April 6 and 17, 2020) when there was no information about the possibility of leaving home for training. Participants were assessed by an on-line questionnaire regarding their week training frequency and duration, mood in relation to the confinement and about the use of online training tools before and during the period of confinement by COVID-19. This study was approved by Ethics Committee of the University of Miguel Hernandez (Elche).

### Measures

*Ad hoc* questionnaire was designed in a Likert response format [from nothing (1) to much (5)] to assess the thoughts and feelings these athletes had about confinement or how they perceived this situation to be affecting them (e.g., “I spend a lot of time each day thinking about negative things about my sporting future or what might happen with this season” or “the confinement situation is affecting my preparation as a cyclist”). In addition, questions about how cyclists were conducting their training, both the frequency of training and the duration of the sessions were asked (including different type of training). Finally, the *ad hoc* questionnaire included questions about the use of online training apps both before and during confinement (e.g., are you connecting to virtual or online training platforms during quarantine?).

The evaluation of emotions was carried out with the Positive and Negative Affect Schedule (PANAS) (Watson et al., 1988) in its Spanish adaptation (Sandín et al., 1999). It measures two dimensions: positive affect and negative affect with high reliability indexes (α = 0.89). Positive affect reflects the degree to which a person feels activated and pleasantly engaged while negative affect focuses on unpleasant emotional states (Sandín et al., 1999). The scale is composed of a total of 20 items that describe different emotions and is answered in a Likert response format with scores ranging from 1 (none) to 5 (a lot). In our sample we used an adapted short version of the PANAS of 14 items and obtained a α = 0.75 for positive affect and a α = 0.076 for negative affect.

Complementarily, direct Likert questions about mood perception were asked. Specifically, about Irritability, Fatigue, Energy, support by others, tension, and sadness. Moreover, the perception of difficulty to train was asked.

### Grouping Variables

To compare the use of online training tools (objective 2) and virtual rollers (objective 3) participants were grouped depending on the scores in the following yes/not questions:

• During confinement, do you use some of the following online training tools (facebook, Instagram, and youtube, …)?
• During confinement do you use some of the following virtual training tools (Bkool, Zwift, …)?

### Statistical Analyses

None of the variables were normally distributed, so non-parametric tests were used to accomplish the aims. Thus, to respond to objective 1 (association between the use of online training tools and training frequency/duration and mood during confinement), Spearman correlation analyses were carried out between the perceived use of online tools and the variables of frequency and duration of training, as well as in the mood and difficulties to train during the confinement and perception of the future professional.

To address the 2nd objective (comparison depending on the use of online training tools), participants were classified and compared (Mann–Whitney *U*) in two different ways: (1) according to the use of online training tools before to the confinement; (2), according to start using online training tools during the confinement (in this case, participants that started using online training tools were compared in frequency and duration of training before and during confinement using Wilcoxon test).

For the third objective (effects of virtual roller), sample was divided between those that employed and those that not.

All statistical analyses were performed with the package SPSS 24. Level of significance was established at *p* < 0.05. Effect size is represented with Cohen d.

## Results

First, it is important indicate that all the cyclists in this study had home training tools (mainly rollers) and internet access.

### Relationships Between Online Training Tools Use (Before and During Confinement) and Emotional and Training Variables (Objective 1)

#### Online Training Tools Use Before Confinement

First, cyclists using training apps before confinement continued to use them during confinement (*r* = 0.276, *p* < 0.001).

In addition, the use of online tools before confinement is negatively associated with thinking less negatively about their sporting future during confinement (*r* = −0.116, *p* = 0.036) as well as feeling less irritated (*r* = −0.110, *p* = 0.046). Complementarily, the use of online training tools before confinement is positive and significantly related to positive mood (*r* = 0.146, *p* = 0.008) and negatively to negative mood (*r* = −0.148, *p* = 0.007) during confinement.

#### Online Training Tools Use During Confinement

On the other hand, the use of online training tools during confinement correlates positively with the frequency of training (*r* = 0.119, *p* = 0.001). Regarding the type of training performed, we found that during confinement the higher use of online tools is directly related to the frequency and duration of indoor roller/bike training (*r* = 0.294, *p* = 0.001 and *r* = 0.322, *p* = 0.001, respectively).

In addition, the use of online tools is negatively related to negative thinking about cycling preparation (*r* = −0.113, *p* = 0.042); thus, the more use of online tools during confinement the less the cyclist believes that confinement affects his or her cycling preparedness. Complementarily, the increased use of these tools during confinement correlates negatively with the effort to train (*r* = −0.253, *p* = 0.001). Finally, using training tools is positively related to feeling greater energy (*r* = 0.148, *p* = 0.007).

#### Changes in the Use of Online Training Tools During Confinement

When cyclists changed the use of online training tools during confinement (subtracting the frequency of use before confinement to the use during confinement), it was observed that the greater increase using these tools during confinement the more fatigue was perceived by cyclists (*r* = 0.114, *p* = 0.039). In addition, this increase was associated with a higher frequency of training during confinement (*r* = 0.217, *p* = 0.001) and to the increased frequency of training compared to training before confinement (*r* = 0.147, *p* = 0.007). Finally, the increase of using online training tools also correlates positively and significantly with the frequency of functional training (*r* = 0.117, *p* = 0.034) and roller training (*r* = 0.293, *p* = 0.001) and with the duration of roller training (*r* = 0.308, *p* = 0.001).

### Comparison Between the Use of Online Training Tools: Before and During the Confinement (Objective 2)

#### Use of Online Training Tools Use Before the Confinement

When comparing those who used online training tools and those who did not use them before the confinement (Table 1), differences in positive PANAS (*U* = 14.317 (*Z* = 2.725), *p* = 0.006, *d* < 0.1) and negative PANAS [*U* = 9.953 (*Z* = −2.638), *p* = 0.008, *d* < 0.1] were found. In both cases, those who used online tools had greater positive affect and less negative affect than those who did not use training tools. Furthermore, the training frequency during confinement was also different between groups [*U* = 9,988 (*Z* = −2,596), *p* = 0.009, *d* < 0.1] having higher scores those who used online tools. Specifically, those who used before confinement, did more functional training [*U* = 9,498 (*Z* = −3,256), *p* = 0.001, *d* < 0.1] and roller [*U* = 10,329 (*Z* = −2,204), *p* = 0.028, *d* < 0.1] during quarantine than the other group.

**TABLE 1:** Mean and standard deviation (SD) of cyclists that employed (or not) online training tools before confinement.

	No use of online training tools before confinement		Use of online training tools before confinement	
	Mean	SD	Mean	SD
**Emotions and thoughts**				
Confinement feelings	1.99	1.08	1.91	1.18
The confinement situation is affecting her preparation as a cyclist.	2.38	1.18	2.17	1.09
He spends a lot of time each day thinking negative thoughts about his sports future/this season.	1.25	1.10	0.95	1.09
Irritability	1.10	0.97	0.91	1.02
Fatigue	0.92	0.94	0.83	0.94
Energy	1.95	1.01	2.14	1.14
Supported by others	2.37	1.15	2.18	1.21
Tension	1.18	1.08	1.02	1.14
Sadness	1.35	1.13	1.25	1.18
Initial Training Difficulty	4.58	3.14	4.40	3.34
Current training difficulty	4.57	2.68	4.10	3.16
Positive Affect	20.36	4.69	21.81	4.88
Negative Affect	14.43	4.43	13.14	4.28
**Training variables**				
Week training frequency before confinement	7.77	3.32	7.19	3.63
Week training frequency during confinement	9.34	4.27	8.04	3.79
Functional training weekly frequency	3.09	1.89	2.41	1.65
Roller weekly frequency	4.60	2.16	4.05	2.20
Strenght weekly frequency	1.29	1.59	1.23	1.54
Funcional training weekly duration	1.11	0.52	1.07	0.71
Roller training weekly duration	1.75	0.78	1.67	0.97
Strenght weekly duration	0.64	0.69	0.75	0.90

Moreover, those who used online tools before confinement had lower scores in thinking negatively about their sporting/seasoning future [*U* = 10,046 (*Z* = −2,640), *p* = 0.008, *d* < 0.1] and felt less irritated [*U* = 10,504 (*Z* = −2,069), *p* = 0.039, *d* < 0.1] than those that did no use online tools.

#### Initiation of the Use of Online Training Tools During Confinement

When comparing cyclists who have initiated the use of online training tools during confinement and those who did not use them (Table 2) differences have been found in weekly frequency [*U* = 7,112 (*Z* = 2,740), *p* = 0.006, *d* < 0.1], and duration of roller use [*U* = 7,348 (*Z* = 4,371), *p* = 0.000, *d* < 0.1], during confinement. Those who started using online training tools trained more frequently and with longer duration than those who did not use this method.

**TABLE 2:** Mean and standard deviation (SD) of cyclists that began to use training apps during the confinement compared to those that did not use (nor begin) online training tools.

	No online training tools use		Initial online training tools use	
	Mean	SD	Mean	SD
**Emotions and thoughts**				
Confinement feelings	2.10	1.159	1.89	1.016
The confinement situation is affecting her preparation as a cyclist.	2.50	1.225	2.26	1.140
He spends a lot of time each day thinking negative thoughts about his sports future/this season.	1.34	1.176	1.18	1.050
Irritability	1.17	1.089	1.04	0.851
Fatigue	0.86	0.940	0.97	0.945
Energy	1.82	1.027	2.08	0.997
Supported by others	2.25	1.186	2.46	1.122
Tension	1.33	1.124	1.06	1.041
Sadness	1.50	1.212	1.23	1.048
Initial Training Difficulty	4.62	3.178	4.57	3.131
Current training difficulty	5.25	2.656	3.96	2.588
Positive affect	19.76	4.594	20.89	4.764
Negative affect	14.80	4.809	14.14	4.059
**Training variables**				
Week training frequency before confinement	7.7573	3.57131	7.7632	3.10096
Week training frequency during confinement	8.9320	4.30973	9.6842	4.22239
Functional training weekly frequency	4.15	2.307	4.98	1.951
Roller weekly frequency	4.15	2.307	4.98	1.951
Strenght weekly frequency	1.31	1.810	1.26	1.376
Funcional training weekly duration	1.10	0.515	1.12	0.534
Roller training weekly duration	1.49	0.705	1.96	0.797
Strenght weekly duration	0.59	0.633	0.68	0.744

There were also significant differences in energy [*U* = 6,761 (*Z* = 2,013), *p* = 0.044, *d* < 0.1], and in training difficulty [U = 4,252 (*Z* = −3,531), *p* = 0.001, *d* < 0.1], during confinement. Those who initiated the use of online tools had lower scores in training difficulty and higher energy perception during confinement.

Complementary, frequency and duration of training, pre and during confinement, in participants that started using apps were compared. Results showed a significant increase in the frequency and duration in functional training, roller, and strength training [Frequency: *Z* = −7.483 (*p* = 0.001), *Z* = −9.028 (*p* = 0.001) and *Z* = 2.126 (*p* = 0.034); Duration: *Z* = −3.305 (*p* = 0.001), *Z* = −8.558 (*p* = 0.001), respectively (Cohen’s *d* < 0.1)], showing a general increase in frequency and duration of training workouts in participants that started using online training tools.

### Comparison Between Participants That Used Virtual Roller During Confinement (Objective 3)

Results comparing cyclists that used virtual roller with those that did not used (Table 3), revealed that the first group scored lower in the effects of confinement in their perception of preparedness as cyclists [*U* = 10,989 (*Z* = −2.246), *p* = 0.0025, *d* < 0.1]. Moreover, those cyclists that used virtual roller trained more frequently and with higher duration with roller than those that did not used this tool [*U* = 14.235 (*Z* = 5.365), *p* = 0.001, *d* < 0.1 and *U* = 16.539 (*Z* = −5.820), *p* = 0.001, *d* < 0.1, respectively]. In addition, virtual rollers users had less difficulties to train [*U* = 10.627 (*Z* = −2.663), *p* = 0.008, *d* < 0.1] and to follow training program [*U* = 10.751 (*Z* = −2.604), *p* = 0.001, *d* < 0.1]. Complementarily, they felt high fatigue [U = 14.481 (*Z* = 2.105), *p* = 0.035, *d* < 0.1] but perceived higher support by others [*U* = 14.574 (*Z* = 2.152), *p* = 0.031, *d* < 0.1].

**TABLE 3:** Mean and standard deviation (SD) of cyclists that employed (or not) virtual roller during confinement.

	No virtual roller use		Virtual roller use	
		
	Mean	S.D.	Mean	S.D.
**Emotions and thoughts**				
Confinement feelings	1.94	1.13	1.99	1.09
The confinement situation is affecting her preparation as a cyclist…	2.43	1.19	2.16	1.09
He spends a lot of time each day thinking negative thoughts about his sports future/this season.	1.09	1.08	1.26	1.15
Irritability	0.99	1.01	1.12	0.96
Fatigue	0.78	0.86	1.04	1.03
Energy	1.97	1.04	2.08	1.08
Supported by others	2.19	1.20	2.48	1.13
Tension	1.08	1.13	1.21	1.07
Sadness	1.31	1.14	1.36	1.17
Initial training difficulty	4.67	3.21	4.38	3.21
Current training difficulty	4.79	2.82	3.91	2.87
Positive affect	20.57	4.78	21.20	4.88
Negative affect	13.78	4.46	14.39	4.36
**Training variables**				
Week training frequency before confinement	7.24	3.72	8.02	3.01
Week training frequency after confinement	8.32	4.56	9.74	3.46
Functional training weekly frequency	2.76	1.97	2.99	1.74
Roller weekly frequency	3.88	2.51	5.21	1.38
Strenght weekly frequency	1.29	1.66	1.22	1.45
Funcional training weekly duration	1.09	0.61	1.12	0.56
Roller training weekly duration	1.49	0.78	2.04	0.84
Strenght weekly duration	0.64	0.78	0.72	0.77

## Discussion

The main aim of this study was to relate, in professional cyclists, the use of online training tools (including virtual rollers) with frequency and duration of training sessions, as well as with mood and maintenance of daily routines during COVID19 confinement. Results confirmed these significant relationships but with a little effect size and low correlation indexes. Specifically, professional cyclists who use online training tools informed being less pessimistic about their future in comparison with cyclists that do not use these tools. Even using online tools for training before confinement was related to better mood. Therefore, during confinement the use of online training tools would be a recommendable strategy for cyclists to improve mood and their vision of the future. In addition, the increased use of these tools leads to a higher frequency of training, specifically indoor roller and is associated with positive thinking about future preparation. Complementarily, online training tools use was associated with a greater perception of energy. Additionally, an interesting and very useful aspect to highlight is that the use of these online tools was negatively related to the effort that cyclists take to train; so, these methods could be a good instrument to motivate and carry out more effective training routines. In this sense, considering that reduced levels of physical activity have been an issue of concern during COVID-19 confinement (Bentlage et al., 2020) and the effects on mental health as negative affect, well-being and mood, leading to even lower life satisfaction (Ammar et al., 2020a,b,c, 2021a,b), recommending the use of online training tools could solve, partially, some of the confinement issues. In this line, it has been reported a greater acceptance among the population of the use of technologies during this period, so the use of these online resources should be considered as appropriate strategies to maintain physical activity (Ammar et al., 2020c, 2021a).

Our results showed also that the increase in the use of online training was related to the greater frequency and duration of training with the roller. On the other hand, those who used to use these tools before confinement showed a better mood than those who did not use them, as shown by the correlations. Furthermore, that previous use also was related to a more positive thought about their sport future compared to those who do not use them. Finally, those cyclists who started using these training methods in confinement had a higher frequency and duration of weekly training with the roller than those who did not use them. Complementarily, the former perceived more energy and less training difficulty during confinement than the latter. All these results go along the lines of confirming the usefulness of the use of technologies to increase or maintain physical activity at home, although it is important to remember that the effect size is low.

All these results show how specific online cycling trainings can be a very interesting tool in cases of confinement, allowing cyclists to maintain a training routine evaluated by its frequency and weekly duration. Complementarily, the fact that the use of online training tools reduces the difficulty of training and makes it more attractive allow to maintain training routines helping cyclists to maintain their fitness level and competitiveness. In this sense, in other athletes, such as football players, the adaptation of training sessions to the conditions of confinement has already been noted and recommended, stressing that medical and physiological standards should be ensured by adapting training sessions to the new conditions and recommending more research dedicated to the adaptation of safe training sessions during confinement (Eirale et al., 2020). In addition, these instruments are related to a higher energy and mainly to a more positive vision about their sport future and their preparation, directly influencing the motivation to keep on training. This is very important because following physical activity routines influence positive mood, as has been demonstrated during the pandemic (Clemente-Suárez et al., 2020). We consider this result is also very relevant and reiterates the need to expand the use of the different online training tools in sportsman. Therefore, these tools allow athletes to stay active, which could translate into continued healthy habits such as better sleep quality or a higher level of physical activity (Trabelsi et al., 2021). Research indicates that behavioral changes have occurred in the population during the pandemic lockdown, both in physical activity habits (Leon-Zarceño et al., 2021) and altered eating behaviors (Ammar et al., 2020b), highlighting the importance of safe physical activity in times of pandemic (Chtourou et al., 2020).

Another important result from our study is derived from the analysis of the use of virtual roller. To our knowledge this is the first study that focus on virtual roller. Considering that our results showed a significant increase in the frequency and duration of training bouts and less harmful confinement effects (preparedness and less difficulties to train), we consider that virtual roller could be a ground-breaking in the field of cycling. Thus, the wide training possibilities that provide to cyclists and teams but overall, the possibilities to train without depending on the weather or confinement, could make these virtual training tools a competitive advantage. Among other advantages offered by this type of rollers is the real-time interaction between geographically distanced cyclists, the possibility of team training with the presence of all the riders, make up for the impossibility of training on the road through indoor training, knowing the real fitness status with relative precision and planning tests remotely, and especially to offer an attractive stimulus to roller training, which facilitates compliance with the routines established by physical trainers or sports directors.

Therefore, as found in other fields of application (Gershkovich et al., 2020; Oh et al., 2020; Price et al., 2020) and in applications more related to physical activity for health purposes (Leahy et al., 2019; Angosto et al., 2020; Gür et al., 2020; Kim et al., 2020), our results also affect the usefulness of the online training tools for a specific field of sport, cycling. Moreover, considering that our sample also includes professional sportsmen, the results can be applied also in the professional world, which could be extrapolated to other fields of professional sport. Complementarily, although this study is focused about confinement by COVID-19, these results could be useful in areas or countries where weather conditions (rain, snow, and wind, …) often prevent going out to train.

Limitations and strengths. The main important limitation of this study is that all the measures are subjective (self-reported) and obtained from an online questionnaire directly asking cyclists about their perception of frequency and duration of training and perceived mood and feelings. Moreover, the low effect size and low correlations, even significant, obtained in all statistical analyses prevent us to made sounding conclusions. Complementarily, this research would have benefited if we had made explicit the specific applications that the cyclists used, mainly in the case of virtual rollers to identify which tools are the most employed and useful. However, the strengths of this study are that it is a specific population that has not been studied during confinement, even though they are eligible for paying attention since they depend on being able to leave home to train. In addition, another strength is that the data were taken at the time of the confinement, two weeks after the start of the confinement when the future was uncertain.

In conclusion, this study shows the benefits of using specific online training tools in competitive cycling. These benefits could be divided in two: training benefits (higher frequency and duration of training with roller) and psychological benefits (positive vision of the future, perception of better preparation, greater perception of energy and less perceived difficulty in training). Future studies should replicate these results and deepen in the type of applications cyclist use.

## Data Availability Statement

The raw data supporting the conclusions of this article will be made available by the authors, without undue reservation.

## Ethics Statement

The studies involving human participants were reviewed and approved by University Miguel Hernández Ethics Committee. The patients/participants provided their written informed consent to participate in this study.

## Author Contributions

AM-T and EL-Z developed the design of the research. All authors participated in the development of the study. MS-R performed the data analyses. All authors contributed in the writing the first draft and reviewing the final draft. The manuscript has been approved by all authors.

## Conflict of Interest

The authors declare that the research was conducted in the absence of any commercial or financial relationships that could be construed as a potential conflict of interest.
