# Corrigendum: The Use of Online Training Tools in Competition Cyclists During COVID-19 Confinement in Spain

**DOI:** 10.3389/fpsyg.2021.684946

**Published:** 2021-05-20

**Authors:** Antonio Moreno-Tenas, Eva León-Zarceño, Miguel Angel Serrano-Rosa

**Affiliations:** ^1^Department of Behavioral Sciences and Health, Miguel Hernández University, Elche, Spain; ^2^Department of Psychobiology, Faculty of Psychology, University of Valencia, Valencia, Spain

**Keywords:** COVID-19, online training tools, confinement, emotions, cycling psychology

In the original article, there was a mistake in Table 3 as published. Some of the means and standard deviations found in Table 3 were wrong. The corrected Table 3 appears below.

**Table 3:** Mean and standard deviation (SD) of cyclists that employed (or not) virtual roller during confinement.

	**No virtual roller use**		**Virtual roller use**	
	**Mean**	**S.D**.	**Mean**	**S.D**.
**Emotions and thoughts**				
Confinement feelings	1.94	1.13	1.99	1.09
The confinement situation is affecting her preparation as a cyclist.	2.43	1.19	2.16	1.09
He spends a lot of time each day thinking negative thoughts about his sports future/this season.	1.09	1.08	1.26	1.15
Irritability	0.99	1.01	1.12	0.96
Fatigue	0.78	0.86	1.04	1.03
Energy	1.97	1.04	2.08	1.08
Supported by others	2.19	1.20	2.48	1.13
Tension	1.08	1.13	1.21	1.07
Sadness	1.31	1.14	1.36	1.17
Initial training difficulty	4.67	3.21	4.38	3.21
Current training difficulty	4.79	2.82	3.91	2.87
Positive affect	20.57	4.78	21.20	4.88
Negative affect	13.78	4.46	14.39	4.36
**Training variables**
Week training frequency before confinement	7.24	3.72	8.02	3.01
Week training frequency after confinement	8.32	4.56	9.74	3.46
Functional training weekly frequency	2.76	1.97	2.99	1.74
Roller weekly frequency	3.88	2.51	5.21	1.38
Strenght weekly frequency	1.29	1.66	1.22	1.45
Funcional training weekly duration	1.09	0.61	1.12	0.56
Roller training weekly duration	1.49	0.78	2.04	0.84
Strenght weekly duration	0.64	0.78	0.72	0.77

The authors apologize for this error and state that this does not change the scientific conclusions of the article in any way. The original article has been updated.

